# Status of Dentistry

**Published:** 1887-07

**Authors:** G. E Corbin


### Status of Dentistry.

BY G. E CORBIN, M.D., D.D.S.

From the very earliest period of human life upon the surface of the earth mankind must have been subjected to the misfortunes of accident and disease. Long prior to the time of any recorded historic event rude attempts were undoubtedly made to alleviate human suffering. The savages of to-day resort to gyrations, incantations, and other irrational modes of treatment. Ignorant, primitive man, with no written language and no record of prior experiences, could not have done very differently. Man's first conceptions must have related to himself, and his immediate surroundings. His own physical strength must have been his unit, or standard of comparison. Weak and inoffensive members of the animal kingdom were adored, because their skins furnished him with clothing, and their carcasses with food. Ferocious beasts, floods, devastating fires, etc., were evil powers to be dreaded and avoided.

Tornadoes, pestilence, and the whole category of human diseases were also evil powers, and the more to be dreaded because their causes were invisible. The stronger and more courageous members of the human family could, and doubtless did, avert some of these impending calamities; and hence were implored to protect, both from visible and invisible evils. In this manner, very naturally arose a class of mediators between mankind and invisible powers, both good and evil. These early priests were really the most intelligent members of their respective communities, and their opinions were the law of the land. They proclaimed the law, the theology, and the medicine of the period. Just so long as diseases were thought to be the result of the maliciousness of invisible evil powers, just so long medical treatment consisted in prayers, offerings, penance, incantations, etc. Just so soon as human intelligence had so far progressed as to see a connection between diseases and natural causes, the priest had a competitor in the field of medical practice. Comprehending natural laws was a slow process in those early times. Many, very many were the long centuries in which the physician with but little light, groped in much darkness. By slow degrees was unfolded a knowledge of some of the general principles which constitute the rudiments of the sciences of anatomy, physiology, pathology, botany, chemistry, etc. This knowledge acquired, the occupation of the physician justly assumed the position of a distinct profession. By a similar process of irresistible growth was developed a profession of law; and thus it was that the profession of the priest was unavoidably subdivided into the three *specialties* of theology, law, and medicine. It was found that in adjusting the fragments of broken bones, and dealing with various other injuries, in *addition* to the knowledge necessary for the selection and administration of proper medicinal substances, skillful *physical* manipulation is a necessity. Physicians possessing this additional qualification are denominated surgeons; and thus it was, by the process of segmentation, that the science of medicine gave birth to the *specialty* of surgery. Only a few centuries ago there was so little known of the foundation sciences so essential in the practice of the healing art, that any person of fair ordinary ability could easily possess himself of the aggregate knowledge of the period. The fact is far otherwise to-day. Each of the numerous basis sciences is an extensive field in itself. *Each* of these branches occupies the entire time, strength, and ability of one or more teachers or professors in our medical colleges. How absurd, then, to assume that the mere college graduate can possess a minute, an expert knowledge of all, or even of *any* of these branches. Good ability and rigid, thorough application will give him a good fair knowledge of the *general principles* of all. That is the most possible.

The education of the young graduate in medicine, therefore, is simply his primary school training. If this work be thoroughly done, and ambition, strength, and ability possessed, a commendable degree of success can be attained by concentration of effort on any congenial one of the numerous branches or departments of the healing art. A mere mechanic can saw off a mangled leg, but it requires the knowledge of the *surgeon* to *prevent* amputation. Precisely so with the teeth. Ignorance will destroy them; intelligence preserve them. A barber or a blacksmith can *extract* teeth; but because of the fact that they sustain precisely the same vital relation to the general organism that does a mangled leg, precisely the same medical knowledge is called for in a successful preservative treatment of them. The very name of surgeon is derived from two Greek words meaning hand work. To dissect out tumors, to carve suitable flaps in amputations, to adjust fragments of broken bones, and so apply splints as to retain them properly in apposition, makes skillful hand work a necessity; hence, the name surgeon. A far greater accuracy and delicacy of mechanical manipulation is essential in the successful practice of oral surgery, ophthalmic surgery, or dental surgery. These facts prove conclusively that *dental* surgery is just as much a department or specialty of general medicine, as is so called, "general surgery." It requires mechanical skill to execute what trained judgment dictates. Neither can succeed alone. For this reason the "short cut" specialist—he who skips the primary school training—to draw it mildly, can not *entirely* succeed. Because of the extent of the field, specialists have become unavoidable necessities; but they can never become independent of the parent science. The best fruit is found at the extremities of the branches, but a branch disconnected from the trunk can not produce it. A true specialist must as certainly draw his nourishment from the fountain head. An expert specialist can not possess his knowledge and ability independent of, but he must possess it beyond, or in addition to, a fair knowledge of all the basis or parent sciences.

No one can successfully treat any of the numerous diseases of the eye unless fully conversant with its intimate relations to the general organism. Should the ophthalmologist, therefore, seek a degree appropriate to his specialty, it should invariably prove the possession of knowledge in addition to that designated by the usual medical degree. Precisely so with dentistry, or any other specialty of medicine. Probably there is no other specialty of medicine that has attained to honorable recognition as such, in so brief a period of time, as has dentistry; still, to be consistent, to endow it with its proper and just significance, the degree of the dental colleges should always follow—never precede— the attainment of the general medical degree. The time is not far distant, when to attain admission to any reputable dental college, the applicant must be a graduate in medicine.

---

FACTS.—"The high road to truth is the knowledge of facts, and well it is for searchers after truth when facts can be ascertained and carefully recorded."

"Symptoms are the alphabet, cases the language, of disease, and that physician subserves his profession who carefully records his experience."

